# CD8^+^ T cells from patients with narcolepsy and healthy controls recognize hypocretin neuron-specific antigens

**DOI:** 10.1038/s41467-019-08774-1

**Published:** 2019-02-19

**Authors:** Natasja Wulff Pedersen, Anja Holm, Nikolaj Pagh Kristensen, Anne-Mette Bjerregaard, Amalie Kai Bentzen, Andrea Marion Marquard, Tripti Tamhane, Kristoffer Sølvsten Burgdorf, Henrik Ullum, Poul Jennum, Stine Knudsen, Sine Reker Hadrup, Birgitte Rahbek Kornum

**Affiliations:** 1 Department of Health Technology, Section of Experimental and Translational Immunology, Technical University of Denmark, 2800 Kgs Lyngby, Denmark; 2 Department of Clinical Biochemistry, Molecular Sleep Laboratory, Rigshospitalet, 2600 Glostrup Denmark; 3 Department of Clinical Immunology 2034, Copenhagen University Hospital, Rigshospitalet, 2100 Copenhagen, Denmark; 4 Department of Clinical Neurophysiology, Danish Center for Sleep Medicine, Rigshospitalet, 2600 Glostrup Denmark; 5 Norwegian Centre of Expertise for Neurodevelopmental Disorders and Hypersomnias (Nevsom), Department of Rare Disorders, Oslo University Hospital, Ullevål, 0424 Oslo, Norway; 6 Department of Neuroscience, University of Copenhagen, 2200 Copenhagen, Denmark

## Abstract

Narcolepsy Type 1 (NT1) is a neurological sleep disorder, characterized by the loss of hypocretin/orexin signaling in the brain. Genetic, epidemiological and experimental data support the hypothesis that NT1 is a T-cell-mediated autoimmune disease targeting the hypocretin producing neurons. While autoreactive CD4^+^ T cells have been detected in patients, CD8^+^ T cells have only been examined to a minor extent. Here we detect CD8^+^ T cells specific toward narcolepsy-relevant peptides presented primarily by NT1-associated HLA types in the blood of 20 patients with NT1 as well as in 52 healthy controls, using peptide-MHC-I multimers labeled with DNA barcodes. In healthy controls carrying the disease-predisposing HLA-DQB1*06:02 allele, the frequency of autoreactive CD8^+^ T cells was lower as compared with both NT1 patients and HLA-DQB1*06:02-negative healthy individuals. These findings suggest that a certain level of CD8^+^ T-cell reactivity combined with HLA-DQB1*06:02 expression is important for NT1 development.

## Abstract (web summary)

Autoreactive T cells are suspected to destroy hypocretin-producing neurons in narcolepsy. Here the authors detect CD8 T cells recognizing narcolepsy-related proteins in healthy individuals and in patients with narcolepsy, and show that the frequency of self-reactive CD8 T cells differs between patients and controls sharing the same HLA-II risk allele.

## Introduction

Narcolepsy type 1 (NT1) is a chronic neurological sleep disorder characterized by dysregulation of the sleep–wake cycle, leading to early occurring rapid eye movement (REM) sleep, excessive daytime sleepiness, and disrupted night time sleep. Another characteristic of NT1 is muscle tonus dysregulation during wakefulness, resulting in sudden loss of muscle tone (cataplexy). Furthermore, sleep paralysis, hypnagogic hallucinations, and REM sleep behavior disorder/REM sleep without atonia are often seen^1–3^. NT1 is caused by disrupted signaling of the sleep-regulating neuropeptide hypocretin in the brain^4^ and it has been shown that this is owing to the loss of specific neurons in the hypothalamus that produce hypocretin^5,6^.

An autoimmune basis for NT1 has long been suspected based on a strong association with the common HLA-DQ haplotype, DQA1*01:02/DQB1*06:02, which encodes the MHC class II DQ0602 heterodimer^7,8^. This HLA association is one of the highest known: up to 98% of NT1 patients with demonstrated hypocretin deficiency carry DQ0602 versus ~25% of the healthy population^7,9^.

Associations between several MHC class I molecules and narcolepsy have also been suggested by two independent studies^10,11^. HLA-A*11:01, HLA-B*51:01, and HLA-C*04:01 were found in both studies, whereas HLA-B*35:01 and HLA-B*35:03 were found in the study by Tafti et al.^10^ and Ollila et al.^11^, respectively; the discrepancy between the two subtypes is likely owing to ethnicity differences in the two cohorts. Ollila et al.^11^ further reported that HLA-B*18:01 is associated with narcolepsy, whereas HLA-B*07:02 had a weak protective effect.

Following the 2009/2010 H1N1 influenza vaccination campaigns with Pandemrix, as well as after the H1N1 epidemic itself, narcolepsy incidence dramatically increased in several countries^12–14^, further substantiating the role of the immune system in NT1 disease development.

Remarkably, even after the discovery of hypocretin-producing neurons as the putative autoimmune target, attempts to demonstrate narcolepsy-associated autoimmune responses have largely been unsuccessful (reviewed in ref. ^15^), until recently where autoreactive CD4^+^ T cells targeting hypocretin were detected in blood samples from narcolepsy patients^16^ and CD4^+^ T cells recognizing hypocretin were demonstrated to cross-react to the hemagglutinin protein from the 2009/2010 H1N1 influenza A virus^17^. As neurons express only MHC class I and not class II molecules under normal physiological conditions^18^, cytotoxic CD8^+^ T cells are the most likely effector cells in the autoimmune destruction of hypocretin neurons^19^. This is supported by the finding of post mortem hypothalamic CD8^+^ T-cell infiltration in a case of NT1 secondary to anti-Ma-associated diencephalitis^20^. The CD8^+^ T-cell infiltration was associated with a complete loss of hypocretinergic neurons. Importantly, it has also been demonstrated in a mouse model that cytotoxic CD8^+^ T cells with reactivity toward hemagglutinin can specifically kill hypocretin neurons if these transgenically express hemagglutinin. This was not the case for CD4^+^ T cells targeting hemagglutinin. Even though these cells infiltrated the brain and caused local inflammation, this did not lead to loss of hemagglutinin-expressing hypocretin neurons^21^. Thus, even though autoreactive CD4^+^ T cells might initiate the disease process, we hypothesize that the presence of autoreactive CD8^+^ T cells could be necessary for the development of genuine NT1. In the recent study by Latorre et al.^16^ describing autoreactive CD4^+^ T cells, the researchers also searched for autoreactive CD8^+^ T cells. This was limited to reactivity toward hypocretin, and only 10 NT1 patients and 9 healthy controls were tested. Three of these patients, and two controls harbored CD8^+^ T cells responsive toward hypocretin epitopes. Even though hypocretin is the best-known marker of hypocretin neurons, other proteins specific to these neurons could also be the target of an autoimmune process. Testing for CD8^+^ reactivity toward other targets in NT1 blood samples is therefore an important next step for understanding NT1 pathogenesis.

We use a recently developed technique for detection of antigen specific CD8^+^ T cells that is especially valuable in identifying CD8^+^ T-cell populations of low frequency and affinity, as it does not rely exclusively on fluorescence separation like conventional tetramer methods^22^. Instead, it utilizes DNA barcode-labeled peptide-MHC (pMHC) complexes to identify CD8^+^ T cells specific for the peptide presented. Using this method, we were able to screen for CD8^+^ T-cell recognition of 1183 peptides expressed in hypocretin neurons and detect such cells in both NT1 patients and healthy controls. We observe a number of differences in the CD8^+^ T-cell recognition profile between NT1 patients and healthy controls expressing HLA-DQB1*06:02 and hypothesize that the combination of a certain level of CD8^+^ T-cell reactivity and HLA-DQB1*06:02 expression is necessary for NT1 development.

## Results

### Experimental strategy and peptide selection

**Fig. 1:**
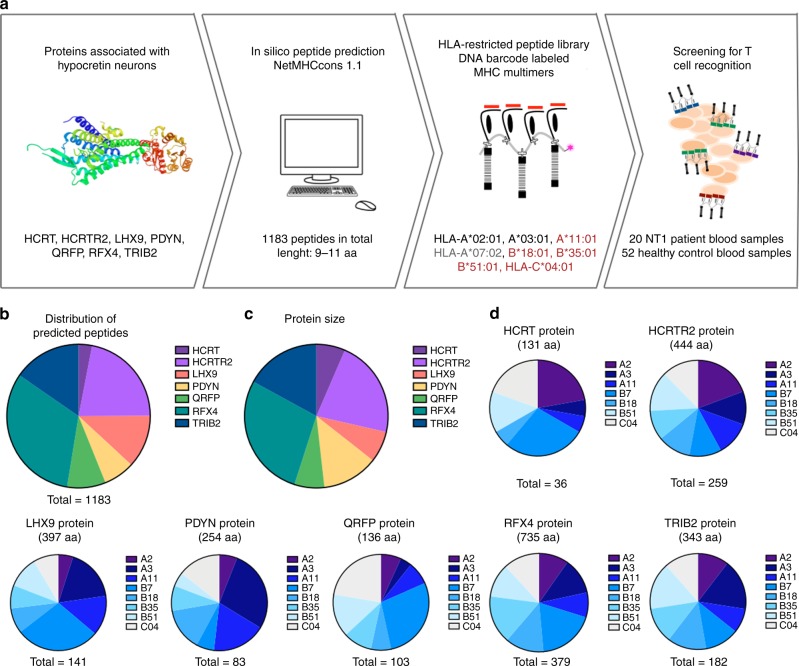
Experiment strategy and peptide prediction. **a** From seven different proteins, peptides with strong binding affinity to eight different HLA types were predicted using the prediction server NetMHCcons1.1. NT1-associated, protective and neutral HLA types are indicated in red, gray and black, respectively. The predicted peptides were used to generate 1183 unique pMHC multimers labeled with DNA barcodes, that were in turn used to screen NT1 patients and healthy controls. **b** All predicted peptides distributed according to protein of origin. **c** The comparative size of the different proteins included. **d** The distribution of the predicted peptides among the eight chosen HLA types within each protein. aa: amino acid. HCRTR2: hypocretin receptor 2. LHX9: Lim homeobox 9. PDYN: prodynorphin. HCRT: hypocretin precursor protein. QRFP: pyroglutamylated RFamide peptide. RFX4: Regulatory factor x4. TRIB2: Tribbles homolog 2

To test the hypothesis that NT1 is mediated by CD8^+^ T cells, we screened blood samples from 20 NT1 patients and 52 healthy controls for the presence of autoreactive CD8^+^ T cells targeting epitopes present in hypocretin neurons. NT1 was diagnosed according to the International Classification of Sleep Disorders, Edition 3 (ICSD-3)^23^. An overview of the experimental strategy is given in Fig. 1a and the core demographical, clinical, and sleep investigation parameters for the narcolepsy cohort are shown in Table 1.

**Table 1:** Demographic, clinical, and paraclinical data of the Narcolepsy cohort

	Narcolepsy type 1 *N* = 20
Gender (male), *n* (%)	9 (45%)
Age (years), mean (range)	25.6 (7–62)
Disease duration (years), mean (range)	5.3 (1–9)
HLA-DQB1*0602- positivity, *n* (%)	19 (95%)
CSF hcrt-1 ≤ 110 pg ml^−1^, *n* (%)	20 (100%)
Daytime sleepiness, *n* (%)	20 (100%) *N* = 20
Epworth Sleepiness Scale, mean ± SEM	16.8 ± 0.5 *N* = 16
Cataplexy presence, *n* (%)	19 (95%) *N* = 20
Hypnagogic hallucinations, *n* (%)	15 (82%) *N* = 19
Sleep paralysis, *n* (%)	11 (62%) *N* = 18
Disrupted night sleep, *n* (%)	17 (87%) *N* = 20
MSLT sleep latency (min.) mean ± SD	3.28 ± 1.98 *N* = 20
SOREMPs (number) mean ± SD	3.95 ± 0.91 *N* = 20

*MSLT* multiple sleep latency test, *SOREMPs* sleep onset REM periods, CSF hcrt-1: hypocretin-1 levels in the cerebrospinal fluid

As nearby neurons in the hypothalamus are not affected in NT1, the epitopes should derive from proteins unique to hypocretin neurons, at least locally but preferably globally. Several studies have aimed to characterize the signature of hypocretin neurons^24–29^. By combining data from these studies and also taking into account the peripheral tissue expression of each target^30^, we selected the following seven, potentially NT1-related proteins for our screening: the precursor protein for the hypocretin peptides (HCRT), the hypocretin receptor 2 (HCRTR2), Lim homeobox 9 (LHX9), prodynorphin (PDYN), the precursor protein for the pyroglutamylated RFamide peptides (QRFP), regulatory factor x4 (RFX4), and tribbles homolog 2 (TRIB2) (details listed in Table 2).

**Table 2:** Overview of the protein targets included in the study and potential targets of interest

Targets included in the study							
Protein	Full name	Size (amino acids)	Type	Relation to hypocretin neurons/narcolepsy	Reference	Allen brain atlas	Peripheral expression^a^
HCRT	Hypocretin neuropeptide precursor	131	Peptide precursor	Uniquely produced in the neurons	^25, 26^	Specific	Very limited
PDYN	Prodynorphin	254	Peptide precursor	Missing post mortem in hypothalamus from NT1.	^25, 26^	Hypothalamus + other brain areas	Very limited
QRFP	Pyroglutamylated RFamide peptide	136	Peptide precursor	Peptide related to sleep regulation	^27^	Only in hypothalamus	Low, gut-intestinal tract
LHX9	Lim homeobox 9	397	Transcription factor	Transcription factor regulating hcrt expression.	^25, 28^	All brain	Very limited
RFX4	Regulatory factor x4	735	Transcription factor	Unknown but highly specific to hcrt neurons	^25^	Only in hypothalamus	Very limited
TRIB2	Tribbles pseudokinase 2	343	Cytosolic protein, pseudokinase	Autoantibodies detected in NT1	^24^	All brain	Yes
HCRTR2	Hypocretin receptor 2	444	Receptor, GPCR	Autoantibodies detected in NT1. HCRTR2 is not expressed in hcrt neurons	^29^	No signal	Low, kidney, heart
**Targets not included in the study **							
NPTX2	Neuronal pentraxin-2	431	Synaptic protein	Missing in hypothalamus from NT1. Also known as NARP	^25, 26^	Hypothalamus + other brain areas	Medium, adrenal gland
IGFBP3	Insulin-like growth factor-binding protein 3	297	Cytosolic protein	Regulates hypocretin expression	^25, 26^	Hypothalamus + other brain areas	High, many organs
PLAGL1	PLAG1 like zinc finger 1	463	Zinc finger protein	Unknown	^24, 25^	Hypothalamus + other brain areas	Medium, many organs
NR6A1	Nuclear receptor subfamily 6 group A member 1	480	Nuclear receptor	Regulates hypocretin expression	^24, 25^	Hypothalamus + other brain areas	Low, many organs

^a^Data from ref. ^30^

Using the NetMHCcons 1.1 in silico prediction algorithm for peptide-MHC class I binding (www.cbs.dtu.dk/services)^31^, we predicted 9- to 11-mer peptides from these seven selected proteins with the ability to bind to eight different HLA-I molecules. The HLA-I molecules were chosen based on reported associations with narcolepsy in two independent studies^10,11^, i.e., HLA-A*11:01, HLA-B*18:01, B*35:01, B*51:01, and HLA-C*04:01. Furthermore, HLA-A*02:01, -A*03:01, and HLA-B*07:02 were also included owing to their high prevalence in the available samples. Peptides with %Rank scores of two or below were defined as binders and included in the study, giving a total of 1183 peptides across the seven different proteins and eight different HLAs.

The distribution of peptides was not equal between the different proteins (Fig. 1b). The number of predicted HLA-binding peptides ranged from 36 in HCRT to 379 in RFX4. This is mostly an effect of the size differences between the proteins, ranging from 131 amino acids (aa) to 735 aa (Fig. 1c). Within each different protein, the number of predicted peptides with high affinity for each HLA also varied (Fig. 1d). For some proteins (RFX4, HCRTR2, and TRIB2), the distribution of peptides was fairly even between the eight different HLA types included, whereas for others (LHX9, PDYN, HCRT, and QRFP) some HLA alleles were over- or underrepresented.

### Screening for T-cell recognition of NT1-related peptides

**Fig. 2:**
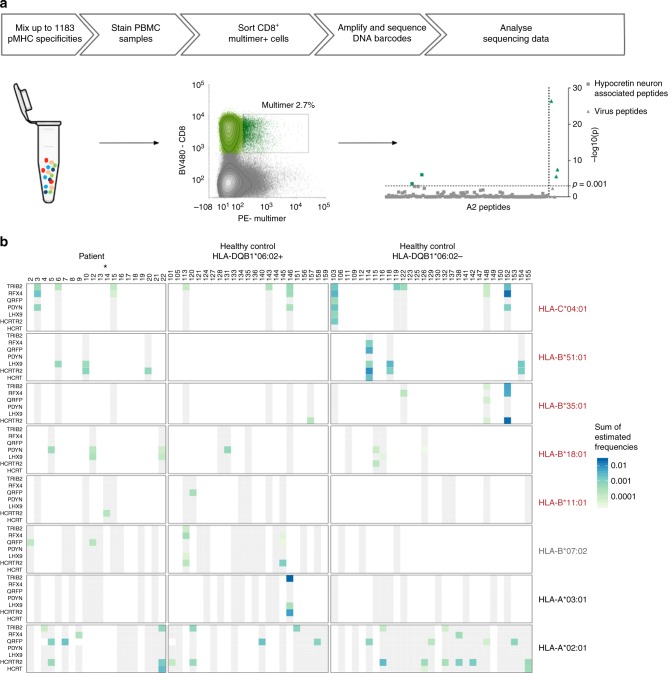
Overview of the experiment pipeline and T-cell screening. **a** Overview of the experiment pipeline. MHC multimers are mixed and used to stain patient and healthy control PBMC samples. CD8^+^, multimer-binding T cells are sorted and associated DNA barcodes are amplified, sequenced, and analyzed. This process is depicted with a sort plot from donor 138 and the CD8^+^ T-cell recognition detected in the given sample. Recognition is defined based on the log_2_fold change of the number of reads compared with triplicate baseline samples with *p* < 0.001 (egdeR). The axis is transformed to –log_10_(*p*) for visualization. –log_10_ (0.001) = 3 (dotted line). **b** Overview of proteins and HLA types included in the screen. Each column represents one donor. Donors are grouped in three cohorts: NT1 patients and HLA-DQB1:06.02-positive and negative healthy controls. Rows show the seven proteins for each of the eight different HLA types. Blue–green color grading determines the detection of CD8^+^ T-cell populations specific for one or more peptides from the protein in the specific HLA context, at levels according to scale. The estimated frequency represents the sum of frequencies for all NT1-related CD8^+^ T-cell populations, in each donor. Gray color coding indicates that the donor carries the given HLA type and was screened for recognition of the given protein in the specific HLA context, but no recognition was detected. White color coding indicates that the donor does not carry the given HLA type and was consequently not screened with peptides in that HLA context. Star denotes the one patient that was HLA-DQB1*06:02 negative. NT1-associated, protective, and neutral HLA types are indicated in red, gray, and black, respectively. pMHC: Peptide-MHC complex. PE: phycoerythrin. BV480: Brilliant Violet 480

The 1183 predicted HLA-binding peptides were synthesized and used to generate individual pMHC monomers using UV-mediated peptide exchange^32^. We then multimerized the pMHC monomers onto a phycoerythrin (PE)-labeled polysaccharide backbone coupled to a DNA barcode that was unique to each specific pMHC (as described in ref. ^22^). This yielded 1183 different pMHC multimers that were mixed according to donor HLA type (Supplementary Table 1) and used to stain the relevant NT1 samples and healthy controls. CD8^+^ T cells with the ability to bind the pMHC multimers were sorted based on a positive PE signal, the associated DNA barcodes were amplified and the specificity of the CD8^+^ T cells could next be revealed by sequencing of the DNA barcodes. This strategy is depicted in Fig. 2a along with an example of a sorting plot from a healthy donor (#138) and the subsequent analysis of the peptide-specific CD8^+^ T-cell populations present in this sample. Gating strategy and control sorting of CD8^−^ T cells with a PE-positive signal is shown in Supplementary figure 1 and 2, respectively.

The 1183 peptides included in this study are ligands of eight different HLA molecules. The eight HLA molecules cover the HLA haplotypes of the donor cohort for at least one and up to four HLA molecules per donor. All donors were screened only with the fraction of the library that matched their HLA type. For each protein and HLA type, Fig. 2b shows whether a given sample was screened (gray) or not (white) and whether CD8^+^ T cells specific toward peptides from the given protein in the given HLA-I molecule were detected (green/blue color gradient). We estimated the frequency of all multimer-positive CD8^+^ T-cell populations (as previously done in ref. ^22^) as the fraction of sequencing reads specific for a given pMHC out of total reads, multiplied by the percentage of sorted multimer-binding CD8^+^ T cells out of total CD8^+^ T cells. The color gradient in Fig. 2b indicates the estimated frequency of the given multimer-positive population, if only one, or the sum of frequencies if more than one peptide-specific CD8^+^ T-cell population was detected in a sample. A full overview of all the multimer-positive populations found in each sample and the peptide sequences that they each recognize is given in Supplementary Figure 3. As can be seen from Fig. 2b, we observed CD8^+^ T-cell recognition of NT1-related peptides in both NT1 patients and healthy controls. All but one patient carried the NT1-associated HLA class II allele DQB1*06:02, whereas only 23 healthy controls out of 52 carried this allele. For this reason, the healthy controls were divided into two groups depending on their expression of this HLA molecule (Fig. 2b).

### Enhanced T-cell recognition in NT1 patients

**Fig. 3:**
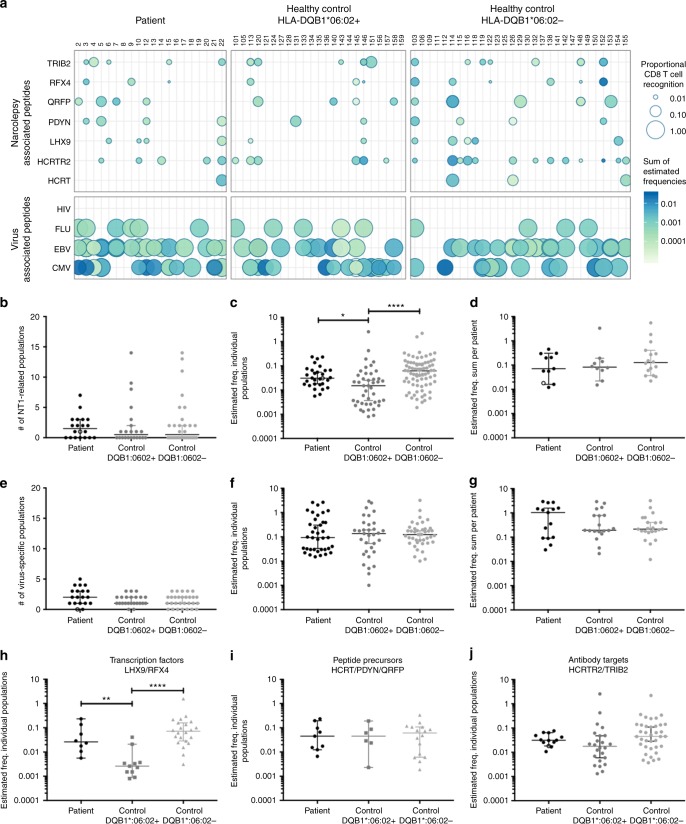
Proportional CD8^+^ T-cell recognition and estimated frequency of detected NT1-related CD8^+^ T-cell populations**. a** Representation of the detected CD8^+^ T-cell recognition in the cohort, of NT1-relevant proteins (upper panel) and known virus epitopes (lower panel). Each dot represents the sum of all detected CD8^+^ T-cell populations with specificity towards a given protein for each individual donor. The size of the dot represents the number of peptides recognized by the given donor, relative to the total number of peptides screened for that donor. The color gradient represents the estimated frequency of the sum of NT1-related CD8^+^ T-cell populations for each donor. **b** The number of NT1-related CD8^+^ T-cell populations detected in each donor. **c** The estimated frequency of each detected CD8^+^ T-cell population, *p* = 0.0124 (*) and *p* < 0.0001 (****) for comparisons between patients and HLA-DQB1*06:02-positive controls and between HLA-DQB1*06:02-positive and negative controls, respectively. (One-way ANOVA and Tukey’s multiple comparisons test on log transformed data). **d** The sum of the estimated frequency for all NT1-related CD8^+^ T-cell populations detected in each donor. **e**, **f**, and **g** show the number of NT1-related CD8^+^ T-cell populations, the estimated frequency of individual populations and the sum for each donor, respectively, for recognition of known virus epitopes. **h**–**j** The estimated frequency of NT1-related CD8^+^ T-cell populations distributed on the different groups of proteins. Related to **h**, *p* = 0.0012 (**) and p < 0.0001 (****) for comparisons between patients and HLA-DQB1*06:02-positive controls and between HLA-DQB1*06:02-positive and negative controls, respectively. (One-way ANOVA and Tukey’s multiple comparisons test on log transformed data). Star **a** or open symbol **b**–**e** denotes the one patient that was HLA-DQB1*06:02 negative. Bars represent median values with 95% CI. Number of donors screened, *n* = 20 for patients, *n* = 23 for HLA-DQB1*06:02 + controls, *n* = 29 for HLA-DQB1*06:02- controls

As some individuals had several CD8^+^ T-cell populations that recognized different peptides within the same protein in the context of more than one HLA type, we summed up the frequencies of all NT1-related CD8^+^ T-cell populations per protein in each individual donor (Fig. 3a, color gradient). As the library size used for T-cell screening depended on the HLA expression of the donor, we determined the proportional CD8^+^ T-cell recognition to correct for any potential biases based on differences in the pMHC library size. The proportional CD8^+^ T-cell recognition was determined as the fraction of peptides recognized by CD8^+^ T cells out of the total number of pMHCs used for screening the individual samples (Fig. 3a, circle size). In addition to the 1183 peptides potentially associated with NT1, all the samples were screened with a panel of known virus epitopes as positive controls. Similar to the NT1-relevant peptides, these were also selected to match the donor’s HLA type. For two healthy donors (#109 and #131), there was no overlap with the viral epitope-HLA complexes available and the HLA haplotype of the donors, and these samples were consequently not analyzed for T-cell recognition of viral antigens. The majority of patients (18/20; 90%) and healthy controls (42/50; 84%) displayed T-cell recognition of virus-derived epitopes (Fig. 3a lower panel). Furthermore, these virus-specific CD8^+^ T-cell populations were generally more frequent than those recognizing NT1-related peptides (Supplementary Figure 4). The virus epitopes were selected from a pool of peptides known to frequently generate T-cell responses in individuals carrying these common virus infections. Hence, the virus epitopes served merely as a positive control and to test for an overall similar immunological recognition capacity between the different cohorts. Consequently, for these highly selected peptides the proportional CD8^+^ T-cell recognition was much higher than seen for the predicted NT1-derived epitopes (Fig. 3a lower panel).

Although T-cell recognition was observed in all three cohorts both toward NT-relevant and virus-derived epitopes, comparison between the three different groups did reveal some interesting immunological characteristics for the NT1-related CD8^+^ T-cell recognition. When counting the number of different peptide-specific CD8^+^ T-cell populations found in each donor in the different cohorts, NT1 patients, HLA-DQB1*06:02-positive controls, and HLA-DQB1*06:02-negative controls, no significant difference was observed based on the average number of CD8^+^ T-cell populations detected (Fig. 3b). However, when evaluating the estimated frequency for each multimer-positive CD8^+^ T-cell population, we observed that the HLA-DQB1*06:02-positive controls had a significantly lower frequency of such T cells compared with both NT1 patients and DQB1*06:02-negative controls. NT1-related CD8^+^ T-cell populations in the latter two groups had estimated frequencies of similar levels (Fig. 3c). When summing-up the frequencies for all different multimer-positive CD8^+^ T-cell populations present in a given donor, there was no difference between the groups (Fig. 3d). We did not observe any differences in the number of protein targets subjected to CD8 T-cell recognition in the three different cohorts. There was no correlation between the duration of disease at the time of T-cell analyses and the number or frequency of NT1-related CD8^+^ T-cell populations detected in the NT1 patients (Supplementary Figure 5).

We analyzed the number and estimated frequency of the virus-specific CD8^+^ T-cell populations to test if the differences observed between the donor cohorts for NT1-related CD8^+^ T-cell recognition also applied to virus-specific CD8^+^ T-cell recognition. For the virus-specific CD8^+^ T-cell populations no differences were observed, neither in terms of number (Fig. 3e) nor estimated frequencies (Fig. 3f, g). For the summed frequencies, there was a tendency toward a higher frequency of virus-epitope recognition in the NT1 patient group, but this was not statistically significant (Fig. 3g).

The observed difference in the estimated frequency of NT1-related CD8^+^ T-cell populations between NT1 patients and HLA-DQB1*06:02-positive controls was more pronounced within certain types of proteins. As is evident from Fig. 3h–j, a significant difference was detected only for transcription factors (LHX9 and RFX4), whereas no difference in CD8^+^ T-cell recognition frequency was observed for the peptide precursor proteins (HCRT, PDYN, and QRFP) or the proteins that have previously been identified as targets for autoreactive antibodies (HCRTR2 and TRIB2).

### T-cell recognition relates to predisposing HLA-I alleles

**Fig. 4:**
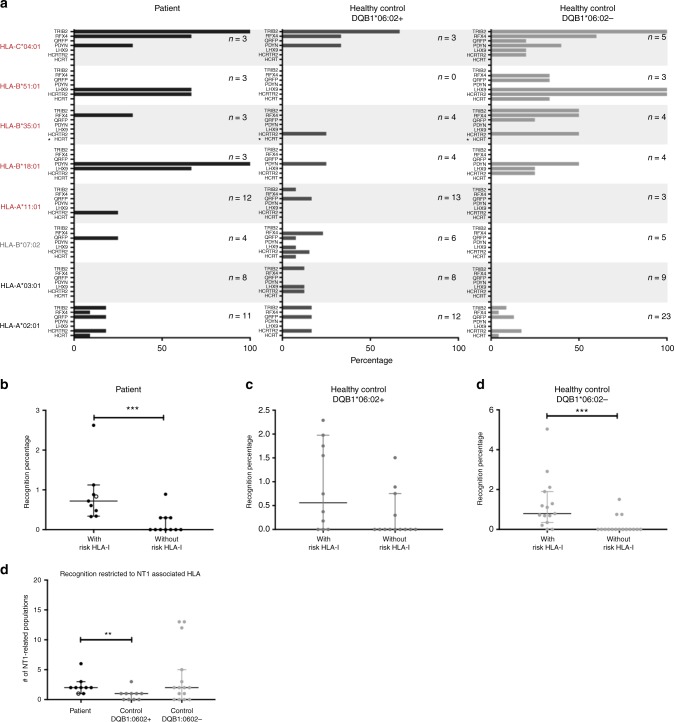
Percentage of donors harboring NT1-related CD8^+^ T-cell populations and recognition percentages. **a** The percentage of donors for each protein-HLA combination with recognition of one or more peptides. *n* = the number of donors that were positive for the given HLA type and thus included in the group. NT1-associated, protective, and neutral HLA types are indicated in red, gray, and black, respectively. Star (*) denotes a protein and HLA combination for which no peptides were predicted to be binders. The fraction of pMHC recognized by T cells out of the total pool of pMHC combinations used to screen the given donor (recognition percentage), is determined for donors with or without a risk-associated HLA-I allele, **b** in the patient cohort, *p* = 0.0004 (***), *n* = 9 and 11 with or without risk HLA-I allele, respectively, **c** in HLA-DQB1*06:02-positive healthy controls, *n* = 10 and 13 with or without risk HLA-I allele, respectively, and **d** in HLA-DQB1*06:02-negative healthy controls, *p* = 0.0003 (***), *n* = 15 and 14 with or without risk HLA-I allele, respectively, (Mann–Whitney test). The recognition percentage for a given donor was calculated as (the number of recognized peptides/total number of peptides used to screen the given patient)*100. **e** The number of CD8^+^ T-cell populations specific toward peptides restricted to an NT1-associated HLA allele, *p* = 0.0041 (**), (Mann–Whitney test). Open symbol denotes the one patient that was HLA-DQB1*06:02 negative. Bars represent median values with 95% CI

We determined the fraction of the individuals in the three different cohorts carrying CD8^+^ T cells specific toward a given NT1-related protein for a given HLA type. Interestingly, the highest percentages of donors harboring NT1-related CD8^+^ T-cell populations were found within the groups of donors expressing NT1-associated HLA-I molecules (Fig. 4a, NT1-associated HLA-I molecules are given in red). The same pattern emerged when counting the number of different CD8^+^ T-cell populations recognizing each protein-HLA combination, normalized to the total number of possible peptide-HLA specific populations. The total number of possible peptide-specific CD8^+^ T-cell populations that could be detected was calculated as the number of samples with a given HLA type times the number of peptides from a given protein predicted for this HLA molecule (Supplementary Figure 6). Here again, the highest frequency of recognition was found with the NT1-associated HLA alleles.

Of the 20 patients included in this study, 9 were positive for an NT1-associated HLA allele and this was the case for 10/23 HLA-DQB1*06:02-positive controls and 15/29 HLA-DQB1*06:02-negative controls. Interestingly, NT1-related CD8^+^ T-cell recognition was detected in 9/9 NT1 patients carrying an NT1-associated HLA-I allele, whereas this was only true for 4/11 (36%) of the patients that did not (*p* = 0.005, fisher exact test). This was also observed for HLA-DQB1*06:02-negative controls where recognition was detected in 13/15 (87%) and 3/14 (21%) donors with and without an NT1-associated HLA-I allele, respectively (*p* = 0.0007, fisher exact test). No significant difference was observed for the HLA-DQB1*06:02-positive controls. It should be noted, however, that donors carrying an NT1-associated HLA allele generally matched more of the HLA class I alleles that we included in our study, than donors not carrying an NT1-associated HLA allele. Consequently, more peptides were evaluated for T-cell recognition in the individuals having an NT1-associated HLA-I allele, on average 399 versus 246 peptides. To account for this difference, the number of peptide-specific CD8^+^ T-cell populations in each donor was normalized to the total amount of peptides used to screen a given donor. The recognition percentage for patients (Fig. 4b) and HLA-DQB1*06:02-negative controls (Fig. 4d) was significantly different when stratifying the donors according to their expression of NT1-associated HLA class I alleles, whereas this was not the case for HLA-DQB1*06:02-positive controls (Fig. 4c).

Looking closer at only the CD8^+^ T-cell recognition of peptides that were restricted toward an NT1-associated HLA allele, we observed that the number of detected NT1-related CD8^+^ T-cell populations differed between the three different donor groups, with HLA-DQB1*06:02-positive controls having significantly fewer of such T-cell populations than NT1 patients and HLA-DQB1*06:02-negative controls (Fig. 4e). As such, 7/9 NT1 patients with NT1-related CD8^+^ T-cell recognition, recognized more than one peptide restricted to an NT1-associated HLA allele, whereas this was only the case for 1/6 of the HLA-DQB1*06:02-positive healthy controls. Thus, in this control group 5/6 donors harbored only one NT1-related CD8^+^ T-cell population restricted to NT1-associated HLA types.

### Ex vivo and in vitro verification of CD8^+^ T-cell specificity

The detected CD8^+^ T-cell populations with specificity toward NT1-related peptides were, in general, of very low frequency and therefore challenging to detect using conventional fluorescently-labeled MHC multimers, in part owing to limited amounts of sample material. However, an NT1-related CD8^+^ T-cell population recognizing a peptide from LHX9 was detected and confirmed with MHC multimer staining (Supplementary Figure 7a). It was not possible to further in vitro culture and expand these NT1-related CD8^+^ T cells, although virus-specific T cells from both patients and healthy controls could be expanded (Supplementary Figure 7b, c).

### Immunogenic hotspots in NT1-relevant proteins

We mapped the location of the possible NT1-related T-cell recognition observed in this study, both for patients and healthy controls. Looking at each protein included in the study, a series of immunological hotspots seems to be present in these proteins, characterized by the colocalization of peptide sequences of different HLA restrictions that were identified as immunogenic across the cohort (Supplementary Figure 8).

## Discussion

Here we present a comprehensive screening for CD8^+^ T-cell recognition of NT1-relevant proteins in peripheral blood mononuclear cells (PBMCs) from well-characterized hypocretin-deficient NT1 patients and healthy controls.

We used DNA barcode-labeled MHC multimers to screen for CD8^+^ T-cell recognition of a large library of 1183 NT1-relevant peptides. These peptides were restricted to a range of HLA molecules (HLA-A*11:01, HLA-B*18:01, B*35:01, B*51:01, and HLA-C*04:01) based on their previously described association with NT1 and three other HLA alleles (HLA-A*02:01, -A*03:01, and HLA-B*07:02) based on their high prevalence in the available samples. The samples were divided into three cohorts: NT1 patients (all but one being HLA-DQB1*06:02-positive), HLA-DQB1*06:02-positive healthy controls, and HLA-DQB1*06:02-negative healthy controls. We observed the expected levels of CD8^+^ T-cell recognition of virus-derived epitopes in all three cohorts; no differences were observed in virus recognition frequency or magnitude. In all three cohorts, recognition of possible NT1-associated peptide epitopes was observed, although at a significantly lower level than for virus-derived epitopes. Interestingly, a number of differences in the CD8^+^ recognition profile were observed between NT1 patients and the cohort of healthy controls also expressing HLA-DQB1*06:02. We found the estimated frequency of individual NT1-related CD8^+^ T-cell populations to be significantly higher in NT1 patients compared with HLA-DQB1*06:02-positive healthy controls. Subgrouping the protein targets revealed that this difference was evident only for the transcription factors LHX9 and RFX4, indicating that these may be important targets for disease development. Furthermore, within the patient cohort, CD8^+^ T-cell recognition was observed to a significantly higher fraction of the tested NT1-related peptides (recognition percentage) in the patients carrying NT1-associated HLA class I alleles (HLA-A*11:01, HLA-B*18:01, B*35:01, B*51:01, and HLA-C*04:01) compared with the patients that did not. Importantly, such a difference was not observed for HLA-DQB1*06:02-positive healthy controls. Finally, the NT1 patients carried a higher number of CD8^+^ T-cell populations restricted toward NT1-associated HLA types than the HLA-DQB1*06:02-positive controls. This finding supports the role of such HLA-I restriction in disease development and indicates that the presence of these risk-associated HLA class I alleles may enhance the level of CD8^+^ T-cell recognition of NT1-related peptides, even affecting those presented by non-risk HLA molecules.

Strikingly, the enhanced level of T-cell recognition observed in NT1 patients compared with healthy controls expressing HLA-DQB1*06:02 was not evident when compared with HLA-DQB1*06:02-negative healthy controls. In fact, for most of the analyses, this cohort resembled the patient cohort. This finding indicates a role for the level of autoreactive CD8^+^ T cells, both in terms of the number and estimated frequency of individual peptide-specific populations, combined with HLA-DQB1*06:02 expression as important parameters for development of NT1. It could be speculated that the lack of HLA-DQB1*06:02 expression allows the existence of NT1-associated CD8^+^ T-cell recognition without disease development, and that the lower level of CD8^+^ T-cell recognition in the presence of HLA-DQB1*06:02 expression prevents disease development. Thus, the role of HLA-DQB1*06:02 in disease initiation should be further elucidated. It is possible, that autoreactive CD4^+^ T cells, which have so far almost exclusively been detected in NT1 patients and not healthy controls, are necessary for the function of the autoreactive CD8^+^ T cells.

In the recent study by Latorre et al.^16^, detecting NT1-relevant autoreactive CD4^+^ T cells, only controls expressing HLA-DQB1*06:02 were included. Thus, whether the same differences between HLA-DQB1*06:02-positive and negative controls are evident for autoreactive CD4^+^ T cells can only be speculated.

Latorre et al.^16^ detect very limited CD8^+^ T-cell reactivity in both patient and healthy donor cohorts, when screening for T-cell reactivity toward HCRT. Here, we included a broader spectrum of seven proteins with preferential expression in hypocretin-producing neurons. Although we also observe limited CD8^+^ T-cell recognition toward HCRT (in one patient and three HLA-DQB1*06:02-negative controls), the CD8^+^ T-cell recognition was substantially larger for the total pool of proteins. The methods used in the two studies differ with regards to the type of T cells that can be detected. Whereas Latorre et al.^16^ detected only memory T cells with the ability to proliferate in response to HCRT peptides, we detect all CD8^+^ T cells with the ability to recognize and bind to NT1-relevant peptides. We did not include phenotype markers or functional analyses to our screening and can therefore not determine whether the autoreactive CD8^+^ T cells found in patients and healthy controls will differ in terms of phenotype or functionality.

Another challenge for the present study was the low level of CD8^+^ T-cell recognition, which compromised our ability to further study these autoreactive T cells by tracking and expanding them, e.g., using fluorescently labeled MHC multimers. Consequently, functional analysis of the autoreactive CD8^+^ T cells that we detected was not possible in our study, although it would have been very interesting to investigate whether any differences between NT1 patients and healthy controls would have been detected. Furthermore, autoreactive T cells are likely to have lower affinity for their target pMHC than virus-specific T cells, consequently making MHC multimer-based detection difficult. As mentioned earlier, we have previously demonstrated that the DNA barcoding-based strategy is especially valuable for detecting low-affinity T cells^22^, and hence the preferable choice in this case. Despite these limitations, in one case we were able to detect NT1-relevant CD8^+^ T cells by a direct MHC multimer staining, demonstrating the presence of such T cells.

A potential limitation in the present study is the fact that patient samples were collected from 1 to 9 years after disease onset. It is possible, that the autoimmune response in NT1 is not strong enough to establish a long-lasting memory response in all patients. We did not observe a correlation between the time from NT1 diagnosis and the number or frequency of detected NT1-related CD8^+^ T-cell populations, but it is possible that most CD8^+^ T-cell recognition toward hypocretin neurons, will fade over time and be undetectable after 1 year. Hence, screening samples from patients very close to disease onset would be a very interesting next step.

In addition to the finding of autoreactive CD8^+^ T cells in NT1 patients, the present study suggests that low levels of autoreactive CD8^+^ T cells of relevance for NT1 can be detected in most individuals, independent of their disease status and HLA types. Similar observations have been made in several studies of other autoimmune diseases. Disease relevant autoreactive CD8^+^ T cells are found to be present in both multiple sclerosis (MS) patients and healthy controls in several studies of MS^33,34^ and the same is true for type 1 diabetes^35–37^. This points to the presence of an external trigger for the activation of autoreactive CD8^+^ T cells as necessary for disease development. In NT1, external triggers, e.g., H1N1 infection or vaccination, could be hypothesized to boost preexisting autoreactive CD8^+^ T cells.

In a recent study of autoreactive CD8^+^ T cells relevant for type 1 diabetes, the frequency and functional capacity of these cells was found to be equal between diabetes patients and healthy controls. What did, however, distinguish these two donor cohorts was the presence of autoreactive CD8^+^ T cells inside the target organ, which was found mainly in the diabetes patients^36^. In the present study we only investigated the presence of NT1-relevant autoreactive CD8^+^ T cells in blood samples from patients and controls, but this may not reflect the true level and distribution of CD8^+^ T-cell recognition relevant for NT1. Detection of such T cells in the cerebrospinal fluid (CSF) could be interesting and potentially more relevant as a measure of disease-initiating T cells. In the study by Latorre et al.^16^, detection of autoreactive T-cell clones in the CSF was attempted for a few NT1 and NT2 patients, but no such CD4^+^ or CD8^+^ T-cell clones were detected in NT1 patients or controls. Two HCRT reactive CD8^+^ T-cell clones were, however, detected in the CSF from an NT2 patient who was recently diagnosed, again underlining the potential importance of screening for T-cell recognition closer to disease onset.

Some HLA class II DQB1 alleles have been suggested to be protective in NT1. These are 05:01, 06:01, 06:03, and less strong all DQB1*02 alleles. We speculated whether the presence of these alleles differed between controls with or without CD8^+^ T-cell recognition. Only 7 of the 23 HLA-DQB1*06:02-positive controls carried a putatively protective HLA class II type. Three of these showed multimer-positive CD8^+^ T cells. Among the remaining HLA-DQB1*06:02-positive controls that were not protected by their HLA class II type, nine individuals still had multimer-positive CD8^+^ T cells. Furthermore, six of the 20 NT1 patients in this cohort carried a protective HLA class II type, indicating that the protective effect of these HLA types is most likely only minor.

In this study, we find that NT1 patients have enhanced CD8^+^ T-cell recognition of NT1-related proteins compared with HLA-DQB1*06:02-positive healthy controls, both in terms of the frequency and the breadth of their CD8^+^ T-cell recognition. Although the observation of a higher level of autoreactive CD8^+^ T cells in patients, compared with HLA-DQB1*06:02-positive healthy controls, points to some involvement of CD8^+^ T cells in disease development, the autoimmune nature of NT1 is still not indisputably proven. CD8^+^ T cells reside in a complex interplay between many different cells of the immune system, and further studies are needed to fully elucidate the etiology of NT1.

## Methods

### Patient and healthy control material

Twenty ICSD-3^23^ diagnosed Type 1 narcolepsy patients (age range: 7–62 y, mean age: 25.6 y; 9 male) were selected for blood donation from the known patient cohort at the Danish Centre for Sleep Medicine. Inclusion criteria were hypocretin deficiency (CSF hcrt-1 < 110 pg ml^−1^) and disease duration ≤ 10 years (Table 1). Of this patient cohort, 19/20 were HLA-DQB1*06:02-positive. Patients were excluded in cases of: secondary narcolepsy; pregnancy; neurological, or autoimmune co-morbidity (hay fever and asthma were accepted); severe medical or psychiatric co-morbidity; medical contraindications to blood donation; treatment with immunosuppressing drugs at any point in the past 3 months (asthma medication was accepted); or signs/history of infection in the 2 weeks prior to the blood donation.

Two patients were drug naive, 18/20 were using stimulants (methylphenidate, modafinil) and/or anticataplectic drugs (tricyclic antidepressants (TCAs), Venlafaxin (SSRI), or Xyrem (Sodium Oxybate)). Three out of 20 patients were using asthma medication (antihistamines and/or mild inhalator/oral steroids or Montelukast (leukotriene D4 receptor antagonist)). All patients had normal white and red blood cell counts.

Fifty-two healthy controls were selected from participants in the Danish Blood Donor Study based on age and gender; 23 of them were HLA-DQB1*06:02-positive (age range: 28–60 y, mean age: 42 y; nine male), and 29 of them were HLA-DQB1*06:02-negative (age range: 19–65 y, mean age: 40 y; 14 male). Inclusion criteria were age ≥ 18 years and fulfilling the blood bank’s general criteria for health.

### PBMC extraction

To collect PBMCs, a blood donation (450 ml) was obtained via the blood bank of Rigshospitalet, Denmark. The blood donations from the Danish blood bank were transported to the laboratory the same day and PBMCs were fractionated into plasma, buffy coat, and red blood cells. PBMCs from the buffy coat were next extracted using Ficoll gradient reagent, frozen in 10% dimethyl sulfoxide (DMSO) (Sigma) in fetal bovine serum (Gibco), and cryopreserved at −140 °C until use.

### Peptide prediction analysis

The online available prediction algorithm NetMHCcons 1.1 (http://www.cbs.dtu.dk/services/NetMHCcons/), was used to predict 9–11-mer MHC class I T-cell epitopes within seven different proteins associated with hypocretin neurons. Peptides were predicted from the following proteins: HCRT (O43612–1), TRIB2 (3 variants Q92519–1, F8WA18, B5MCX4), RFX4 (10 variants Q33E94–1, Q33E94–2, Q33E94–3, Q33E94–4, R4GMS3, F8VRD4, F8VZC4, B4DZB7, F8W1T9, F8VX50), PDYN (P01213–1), LHX9 (8 variants Q9NQ69–1, Q9NQ69–2, Q9NQ69–3, Q9NQ69–4, H0YL54, H0Y330, A0A087X083, A0A0C4DGY4), QRFP (P83859–1), and HCRTR2 (O43614–1). Eight different HLA molecules were included in the analysis: HLA-A*02:01, HLA-A*03:01, HLA-B*07:02, HLA-A*11:01, HLA-B*18:01, HLA-B*35:01, HLA-B*51:01, and HLA-C*04:01. This yielded a total of 1183 predicted T-cell epitopes spread on the eight different HLA molecules and seven different proteins. The peptides were synthesized and purchased from Pepscan (Pepscan Presto, The Netherlands) and dissolved to 10 mm in DMSO.

### HLA analysis across the cohort

All donors included in the study were HLA typed by next-generation sequencing of the full exome of MHC class I HLA-A, -B, and -C. The full exome of MHC class II DQA was also sequenced, but only exon 2 and 3 of HLA-DQB1 were sequenced (GenDx, Netherlands). MiSeq from Illumina was used for all sequence analyses. The full HLA type of each donor is presented in Supplementary Table 1. The highlighted HLA subtypes are associated with an increased risk of NT1^10,11^.

### Generation of DNA barcodes and dextran conjugation

Oligonucleotides containing distinct 25mer nucleotide sequences (from Xu et al. 2009^38^) were purchased from LGC Biosearch Technologies (Denmark). All oligos carried a 6-nt unique molecular identifier^39^. Oligonucleotides modified with a 5′ biotin tag (oligo A) were joined to unmodified, partially complementary oligonucleotides (oligo B) to generate > 1000 unique double-stranded AxBy DNA barcodes. Combinations of A and B oligos (one of each) were mixed with 5 × Sequenase Reaction Buffer mix (PN 70702, Affymetrix) to final concentrations of 26 μm (Oligo A) and 52 μm (Oligo B), respectively; heated to 65 °C for 2 min; and allowed to anneal by cooling slowly to < 35 °C over 15–30 min. The annealed oligo As and Bs were elongated to create double-stranded AxBy DNA barcodes by adding Sequenase polymerase (70775Y, Affymetrix), 20 μm DTT and 800 μM or 72 μm dNTPs, followed by incubation for 5–10 min at RT. Elongated AxBy barcodes were diluted in nuclease-free water + 0.1% Tween to 2.17 μm (with respect to the A oligo) and stored at −20 °C.

Attachment of 5′ biotinylated AxBy DNA barcodes to PE- and streptavidin-conjugated dextran (provided by Immudex, Denmark) was performed by mixing the two components at final concentrations of 14 × 10^–8^ m dextran backbone and 2.8 × 10^−5^ m barcode in order to obtain ~ 2 barcodes for each dextran backbone and subsequent incubation for 30 min at 4 °C^22^.

### Generation of pMHC-I multimers labeled with DNA barcode

A total of 1183 pMHC complexes were generated through UV-mediated conditional ligand exchange^32^. In short, MHC monomers carrying UV-sensitive ligands were mixed with matching peptides at a final concentration of 50 μg ml^−1^ monomer and 100 mm peptide and exposed to UV light for 60 min. The pMHC complexes were then coupled to DNA barcode- and PE-labeled dextran backbones to a final concentration of 35 μg ml^−1^ monomer and 4.2 × 10^−8^ m barcode- and PE-labeled dextran backbone and incubated for 20 min on ice. Finally a 15× freeze buffer was added to reach phosphate-buffered saline (PBS) + 0.5% bovine serum albuin (BSA) + 100 μg mL^−1^ herring DNA + 2 mm ethylenediaminetetraacetic acid (EDTA) + 5% glycerol and 909 nm d-biotin and after 20 min on ice, the pMHC multimers were stored at −20 °C until use.

### T-cell staining with barcode-labeled MHC multimers

Cryopreserved cells were thawed, washed twice in Roswell Park Memorial Institute (RPMI) + 10% fetal calf serum (FCS) and then washed in barcode-cytometry buffer (PBS + 0.5% BSA + 100 μg mL^−1^ herring DNA + 2 mm EDTA). On the day of staining, barcode-labeled MHC multimers were thawed on ice, centrifuged for 5 min at 3300×*g*, and 1.5 μl (corresponding to 0.043 μg) of each distinct pMHC was carefully taken from the top of each well, to avoid any potential aggregates, and pooled according to the HLA type of the donors. The volume of the reagent pool was reduced by ultrafiltration to obtain a final volume of ~ 80 μL of MHC multimers. Centrifugal concentrators (Vivaspin 6, 100,000 Dalton, Sartorius) were saturated with BSA before use. Following ultrafiltration, the pool of multimers was spun at 10,000×*g* for 2 min to sediment potential aggregates. An aliquot of ~5 μl of the MHC multimer reagent pool was stored at −20 °C for baseline analysis. Prior to staining with the MHC multimers, 50 nm dasatinib was added to up to 10 × 10^6^ cells. The MHC multimer pool was then added to the cells and these were incubated for 15 min at 37 °C in a total volume of 80 μl. Following incubation, the cells were stained with an antibody mix containing CD8-BV480 (BD 566121, clone RPA-T8) (final dilution 1/50), dump channel antibodies (CD4-FITC (BD 345768) (final dilution 1/80), CD14-FITC (BD 345784) (final dilution 1/32), CD19-FITC (BD 345776) (final dilution 1/16), CD40-FITC (Serotech MCA1590F) (final dilution 1/40), and CD16-FITC (BD 335035) (final dilution 1/64)), and a dead cell marker (LIVE/DEAD Fixable Near-IR; Invitrogen L10119) (final dilution 1/1000) and incubated for 30 min at 4 °C. Cells were washed three times in barcode-cytometry buffer and fixed in 1% paraformaldehyde (PFA) for 0.5–24 h before they were washed twice and resuspended in barcode-cytometry buffer. Cells were acquired within a week after multimer staining.

### Sorting of MHC multimer-positive T cells

Multimer-binding CD8 T cells were sorted on a FACSAriaFusion (BD) into BSA saturated tubes containing 100 μl of barcode-cytometry buffer. Using FACSDiva software, we gated on single, live, CD8-positive, and dump channel (CD4, 14, 16, 19, and 40)-negative lymphocytes and sorted all multimer-positive PE cells within this population. The gating strategy is exemplified in Supplementary Figure 1. As tested and described in Bentzen et al.^22^, inclusion of CD8-positive, multimer negative cells in the sorting gate does not have an impact on the final results because the fluorescence signal is used only for sorting out the relevant cells. Determination of antigen specificity is done solely based on the DNA barcode. The sorted cells were centrifuged for 10 min at 5000×*g* and the buffer was removed. The cell pellet was stored at −80 °C in a minimal amount of residual buffer (< 20 μL).

### DNA barcode amplification

DNA barcode amplification was performed using Taq PCR Master Mix Kit (Qiagen, 201443) and 3 μm of forward and reverse primers (LGC Biosearch Technologies)^22^. PCR amplification was conducted on sorted multimer-binding T cells (in < 20 μL of buffer) and on the stored aliquot of the MHC multimer reagent pool (diluted 50,000 × in the final PCR) under the following conditions: 95 °C 10 min; 36 cycles: 95 °C 30 s, 60 °C 45 s, 72 °C 30 s, and 72 °C 4 min. The multimer reagent pool was used as the baseline to determine the number of DNA barcode reads within a non-processed MHC multimer reagent library. PCR products were purified with a QIAquick PCR Purification kit (Qiagen, 28104) and the amplified DNA barcodes were sequenced at Sequetech (USA) using an Ion Torrent PGM 316 or 318 chip (Life Technologies).

### Processing of sequencing data from DNA barcodes

Sequencing data were processed by the software package “Barracoda”, available online at (http://www.cbs.dtu.dk/services/barracoda)^22^. This tool identifies the barcodes used in a given experiment, assigns sampleID and pMHC specificity to each barcode, and counts the total number of reads and clonally reduced reads for each pMHC-associated DNA barcode. Furthermore, it accounts for barcode enrichment based on methods designed for the analysis of RNA-seq data, implemented in the R package edgeR: specifically, log_2_ fold changes in read counts mapped to a given sample relative to the mean read counts mapped to triplicate baseline samples are estimated using normalization factors determined by the trimmed mean of *M*-values method^22,40^. Barcodes with a log_2_ fold change with a *p* < 0.001, which equals a false discovery rate < 0.1 (estimated using the Benjamini–Hechberg method), and with clonal read counts above 100 were considered to represent true CD8^+^ T-cell recognition. The cutoff of 100 reads was chosen based on evaluation of this particular data set, in order to ensure that background events were not included in the analysis. We have previously tested sorting and sequencing of the CD8 negative, dump channel positive cells and did not detect any CD8^+^ T-cell recognition in this population (Supplementary Figure 2a). Also, the inclusion of high-levels of MHC complexes that were generated with non-matching peptides under UV-induced peptide exchange, hence producing unstable, partly defolded complexes, did not substantially impact the background of the MHC multimer staining (Supplementary Figure 2b)

### Verification of specificity with flow cytometry

A small cohort of 40 selected peptides, to which specific T cells had been detected either in several donors or with a high number of reads in single donors, were used to generate fluorescently-labeled MHC multimers. Again, UV-mediated peptide exchange was used and pMHC complexes were multimerized using streptavidin-conjugated PE (405204, BioLegend), APC (405207, BioLegend), PE-Cy7 (405206, BioLegend), PECF594 (562284, BD), BV421 (563259, BD), BV605 (563260, BD), BV650 (563855, BD), BUV395 (564176, BD), and BUV737 (564293, BD). The final concentrations of monomer and peptide were 100 μg ml^−1^ and 200 nm, respectively. Each pMHC was made in two different colors and mixed before the MHC multimers were stored at −20 °C with 0.5% bovine serum albumin (Sigma Aldrich) and 5% glycerol (Fluka). For ex vivo T-cell staining, samples from cells were thawed as described above and 2 × 10^6^ cells were stained with 1 μl of each pMHC, according to HLA type, for 15 min at 37 °C. Cells were then stained with a 5× antibody mix as described for staining with DNA barcode-labeled MHC multimers. Following two washing steps and fixation in PFA, cells were acquired on an LSR Fortessa (BD). The gating strategy is identical to the one described above and presented in Supplementary Figure 1.

### Enrichment of specific T cells

Six donors (three patients and three healthy controls) with detected NT1-relevant CD8^+^ T-cell recognition were selected for enrichment of specific T cells. PE-labeled MHC multimers were generated in the same fashion as described for the verification. Cells were thawed and washed twice in RPMI + 10% FCS and 1 × 10^6^ cells were taken to a new tube and irradiated at ~ 20 Gy for 6 min and 15 sec. These cells were washed twice and kept for later use as feeder cells. From the remaining cells, 20 × 10^6^ cells were stained with 1 μl per pMHC of the PE-labeled MHC multimers for 60 min at 4 °C. Following staining, the cells were washed twice and stained with α-PE beads (Miltenyi Biotec) for 15 min at 4 °C. The cells where then washed twice again and loaded onto a magnetic column placed in the magnetic field of a MACS separator (Miltenyi Biotec), which captured the cells with a PE label through the α-PE beads. After washing the column twice, the column was removed from the separator and the specific cells were washed into a new tube containing X-vivo media (Lonza) + 5% human serum (HS; Sigma Aldrich). The specific cells were counted, washed, and cultured in X-vivo + 5% HS supplemented with 23.8 U mL^−1^ IL-15 (PeproTech, #200–15–10UG) and 100 U mL^−1^ IL-2 (PeproTech, #200–02–50UG). Approximately 5000 specific cells were cultured with 50,000 feeder cells/well as well as 11,000 CD3/CD28 Dynabeads (Gibco). After 1.5 weeks of culturing, 2 × 10^6^ cells were stained with the above-described fluorescently labeled MHC multimers and acquired on an LSR Fortessa (BD).

### Graph generation and statistical data analysis

Graphs were generated using either GraphPad Prism 7.0d for mac or ggplot2 in R and edited using OmniGraffle version 7.8.1. Statistical analysis of DNA barcoding data was performed using the software package “Barracoda” as described above. Significant differences between the number and frequency of detected NT1-related CD8^+^ T-cell populations in Figs. 3, 4, and Supplementary Figure 4 were calculated in GraphPad Prism 7.0b. For Fig. 3 and Supplementary Figure 4, log transformation of data was performed, and parametric tests used to determine significant differences, whereas for Fig. 4 non-parametric tests were used. Normal distribution was tested with D’Agostino normality test. Fisher exact tests were used for contingency analysis of over- or underrepresentation of detected recognition within the subgroups of donors. The single patient (14) which is HLA-DQB1*06:02 negative is removed from comparisons between patients and HLA-DQB1*06:02-positive controls. For all figures, *: *p* < 0.05, **: *p* < 0.01, ***: *p* < 0.001, ****: *p* < 0.0001.

### Ethical approval

All donor material was collected with the approval of the Scientific Ethics Committee of the Capital Region, Denmark (Protocol H-3–2013–054) and written informed consent was obtained according to the Declaration of Helsinki.

### Code availability

All relevant code is available from the authors. For DNA barcode analysis, the tool Barracoda is available online at http://www.cbs.dtu.dk/services/barracoda.

## Supplementary information

Supplementary Figures

## Data Availability

The data that support the findings of this study are available from the corresponding author upon reasonable request.
