# Supplementary material for: CD8+ T cells from patients with narcolepsy and healthy controls recognize hypocretin neuron-specific antigens
Source: Nat Commun. 2019 Feb 19;10:837. doi: 10.1038/s41467-019-08774-1 (PMC6381094; doi:10.1038/s41467-019-08774-1)
Supplement: Supplementary file 1 — Supplementary Figures [file 41467_2019_8774_MOESM1_ESM.pdf]

CD8 T cells from patients with narcolepsy and healthy controls recognize hypocretin neuron-specific antigens

Pedersen et al.

Patients	HLA-A		HLA-B		HLA-C		HLA-DQA1		HLA-DQB1	
**2**	02:01:01	29:02:01	07:02:01	15:01:01	03:04:01	07:02:01	01:02:01	05:01:01	02:01:01	06:02:01
**3**	03:01:01	11:01:01	35:01:01	55:01:01	03:03:01	04:01:01	01:01:01	01:02:01	05:01:01	06:02:01
**4**	01:01:01	02:01:01	07:02:01	08:01:01	07:01:01	07:02:01	01:02:01		06:02:01	
**5**	02:01:01		18:01:01	44:02:01	05:01:01	07:01:01	01:02:01	03:03:01	03:01:01	06:02:01
**6**	03:01:01	68:01:02	35:01:01	51:01:01	04:01:01	15:02:01	01:01:01	01:02:01	05:01:01	06:02:01
**7**	02:01:01	24:02:01	07:02:01		07:02:01		01:02:01		06:02:01	
**8**	03:01:01		07:02:01		07:02:01		01:02:01		06:02:01	
**9**	02:01:01		07:02:01	15:01:01	03:04:01	07:02:01	01:02:01	03:01:01	03:02:01	06:02:01
**10**	03:01:01	11:01:01	07:02:01	51:01:01	07:02:01	15:02:01	01:02:01	03:01:01	03:02:01	06:02:01
**12**	03:01:01	25:01:01	07:02:01	18:01:01	07:02:01	12:03:01	01:02:01		06:02:01	
**13**	01:01:01	03:01:01	07:02:01	15:01:01	07:02:01		01:02:01	05:05:01	03:01:01	06:02:01
**14**	11:01:01	24:02:01	40:01:02	52:01:01	03:04:01	12:02:02	01:03:01	03:03:01	03:01:01	06:01:01
**15**	11:01:01	24:02:01	07:02:01	35:01:01	04:01:01	07:02:01	01:02:01	03:01:01	03:02:01	06:02:01
**16**	02:01:01	26:01:01:01	07:02:01	39:01:01	07:02:01	12:03:01	01:02:01	01:03:01	06:02:01	06:03:01
**17**	01:01:01	02:01:01	44:02:01	57:01:01	05:01:01	06:02:01	01:01:01	01:02:01	05:01:01	06:02:01
**18**	01:01:01	02:01:01	08:01:01	44:02:01	05:01:01	07:01:01	01:02:01	05:05:01	03:01:01	06:02:01
**19**	01:01:01	03:01:01	07:02:01		07:02:01		01:02:01	01:04:01	05:03:01	06:02:01
**20**	01:01:01	02:01:01	08:01:01	51:01:01	01:02:01	07:01:01	01:02:01		06:02:01	
**21**	02:01:01	03:01:01	07:02:01	15:01:01	03:04:01	07:02:01	01:02:01	03:01:01	03:02:01	06:02:01
**22**	02:01:01	25:01:01	18:01:01	57:01:01	06:02:01	12:03:01	01:02:01	02:01:01	03:03:02	06:02:01
**Healthy controls**										
**101**	01:01:01	02:01:01	07:02:01	08:01:01	07:01:01	07:02:01	01:02:01	03:02	03:03:02	06:02:01
**103**	03:01:01	23:01:01	35:01:01	44:02:01	04:01:01	07:04:01	01:01:01	01:02:01	05:01:01	06:04:01
**105**	01:01:01	02:01:01	39:06:02	57:01:01	06:02:01	07:02:01	01:02:01	02:01:01	03:03:02	06:02:01
**106**	02:01:01	32:01:01	07:02:01	14:02:01	02:02:02	08:02:01	01:01:02	02:01:01	02:02:01	05:01:01
**109**	25:01:01	29:02:01	18:01:01	44:03:01	12:03:01	16:01:01	01:01:01	02:01:01	02:02:01	05:01:01
**111**	02:01:01		15:01:01	40:01:02	03:04:01		01:02:01	03:01:01	03:02:01	06:04:01
**112**	24:02:01	30:04:01	07:02:01	50:01:01	06:02:01	07:02:01	02:01:01	05:05:01	02:02:01	03:01:01
**113**	11:01:01	23:01:01	07:02:01	35:01:01	04:01:01	07:02:01	01:01:01	01:02:01	05:01:01	06:02:01
**114**	02:01:01	68:01:02	44:02:01	51:01:01	05:01:01	14:02:01	01:03:01	02:01:01	02:02:01	06:03:01
**115**	01:01:01	03:01:01	18:01:01	37:01:01	06:02:01	12:03:01	01:01:01	05:05:01	03:01:01	05:01:01
**116**	02:01:01	03:01:01	15:01:01	18:01:01	03:03:01	05:01:01	05:01:01	05:05:01	02:01:01	03:01:01
**118**	02:01:01	03:01:01	40:02:01	51:01:01	01:02:01	02:02:02	01:03:01	05:01:01	02:01:01	06:03:01
**119**	02:01:01	23:01:01	07:02:01	44:03:01	04:01:01	07:02:01	01:03:01	02:01:01	02:02:01	06:03:01
**120**	02:01:01	11:01:01	39:01:01	44:02:01	07:02:01	07:04:01	01:02:01	02:01:01	03:03:02	06:02:01
**121**	24:02:01	33:03:01	07:02:01	14:02:01	07:02:01	08:02:01	01:01:01	01:02:01	05:01:01	06:02:01
**122**	02:01:01	03:01:01	35:01:01	44:02:01	04:01:01	05:01:01	01:02:01	03:03:01	03:01:01	06:04:01
**123**	01:01:01	02:01:01	08:01:01	40:01:02	07:01:01	14:02:01	01:02:01	05:01:01	02:01:01	06:04:01
**124**	03:01:01		07:02:01		07:02:01		01:02:01		06:02:01	
**125**	02:01:01	29:02:01	38:01:01	44:02:01	05:01:01	12:03:01	01:04:01	05:01:01	02:01:01	05:03:01
**126**	02:01:01	11:01:01	18:01:01	57:01:01	05:01:01	06:02:01	01:03:01	02:01:01	03:03:02	06:01:01
**127**	02:01:01	30:01:01	13:02:01	15:01:01	06:02:01	07:02:01	01:02:01	02:01:01	03:02:01	06:02:01
**128**	03:01:01	25:01:01	18:01:01	40:01:02	03:04:01	12:03:01	01:02:01	01:02:02	05:02:01	06:02:01
**129**	02:01:01	02:05:01	44:02:01	50:01:01	05:01:01	06:02:01	01:02:01	03:01:01	03:02:01	06:09:01
**130**	03:01:01	26:01:01:01	38:01:01	40:01:02	03:04:01	12:03:01	01:02:01	05:05:01	03:01:01	06:04:01
**131**	25:01:01	32:01:01	18:01:01	27:05:02	02:02:02	12:03:01	01:02:01	03:02	03:03:02	06:02:01
**132**	02:01:01	11:01:01	40:01:02	44:02:01	03:04:01	05:01:01	01:02:01	03:03:01	03:01:01	06:04:01
**133**	02:01:01	03:01:01	07:02:01	57:01:01	06:02:01	07:02:01	01:02:01	01:04:01	05:03:01	06:02:01
**143**	03:01:01	11:01:01	07:02:01	40:02:01	02:02:02	07:02:01	01:02:01	05:05:01	03:01:01	06:02:01
**145**	01:01:01	11:01:01	07:02:01	18:01:01	07:01:01	07:02:01	01:02:01	03:01:01	03:02:01	06:02:01
**136**	03:01:01	24:02:01	07:02:01	15:01:01	03:03:01	07:02:01	01:02:01	05:05:01	03:01:01	06:02:01
**137**	02:01:01	11:01:01	44:02:01	56:01:01	01:02:01	05:01:01	01:03:01	03:01:01	03:02:01	06:03:01
**138**	02:01:01	24:02:01	44:02:01	55:01:01	03:03:01	05:01:01	01:01:01	01:02:01	05:01:01	06:04:01
**140**	01:01:01	02:01:01	07:02:01	08:01:01	07:01:01	07:02:01	01:02:01	05:01:01	02:01:01	06:02:01
**141**	02:01:01	03:01:01	14:01:01	14:02:01	08:02:01		01:02:01	03:03:01	03:02:01	06:09:01
**142**	02:01:01	31:01:02	07:06:01	40:01:02	03:04:01	15:05:02	01:03:01	03:01:01	03:02:01	06:03:01
**143**	03:01:01	11:01:01	35:01:01	41:02:01	04:01:01	17:03	01:02:01	03:03:01	03:01:01	06:02:01
**144**	01:01:01	03:01:01	07:02:01	08:01:01	07:01:01	07:02:01	01:02:01	05:01:01	02:01:01	06:02:01
**145**	02:01:01	11:01:01	07:02:01	55:01:01	03:03:01	07:02:01	01:02:01	01:04:01	05:03:01	06:02:01
**146**	03:01:01	25:01:01	18:01:01	35:01:01	04:01:01	12:03:01	01:02:01		06:02:01	
**147**	02:01:01	31:01:02	27:05:02	40:01:02	02:02:02	03:04:01	01:02:01	03:03:01	03:02:01	06:04:01
**148**	02:01:01	11:01:01	35:01:01	40:02:01	02:02:02	04:01:01	01:03:01	03:01:01	03:02:01	06:03:01
**149**	03:01:01	24:02:01	15:01:01	38:01:01	03:04:01	12:03:01	01:03:01	03:01:01	03:02:01	06:03:01
**150**	02:01:01		40:01:02	44:02:01	03:04:01	05:01:01	01:01:01	01:03:01	05:01:01	06:03:01
**151**	02:01:01	24:02:01	40:01:02	44:02:01	03:04:01	16:04:01	01:02:01	05:05:01	03:01:01	06:02:01
**152**	02:01:01	03:01:01	35:01:01	39:01:01	04:01:01	12:03:01	01:02:01	05:05:01	03:01:01	06:04:01
**153**	02:01:01		40:01:02	44:02:01	03:04:01	05:01:01	01:01:01	01:02:01	05:01:01	06:04:01
**154**	02:01:01	31:01:02	40:01:02	51:01:01	03:04:01	15:02:01	01:02:01	03:01:01	03:02:01	06:04:01
**155**	02:01:01	11:01:01	39:06:02	40:01:02	03:04:01	07:02:01	01:02:01	03:01:01	03:02:01	06:04:01
**156**	02:01:01	02:05:01	07:02:01	50:01:01	06:02:01	07:02:01	01:02:01	02:01:01	02:02:01	06:02:01
**157**	02:01:01		35:01:01	44:02:01	03:03:01	05:01:01	01:02:01	03:02	03:03:02	06:02:01
**158**	01:01:01	02:01:01	07:02:01	08:01:01	07:01:01	07:02:01	01:02:01	05:01:01	02:01:01	06:02:01
**159**	02:01:01	02:06:01	15:01:01	40:06:01	03:03:01	08:01:01	01:02:01	01:03:01	06:01:01	06:02:01

Supplementary Table 1
The full HLA type of each donor.

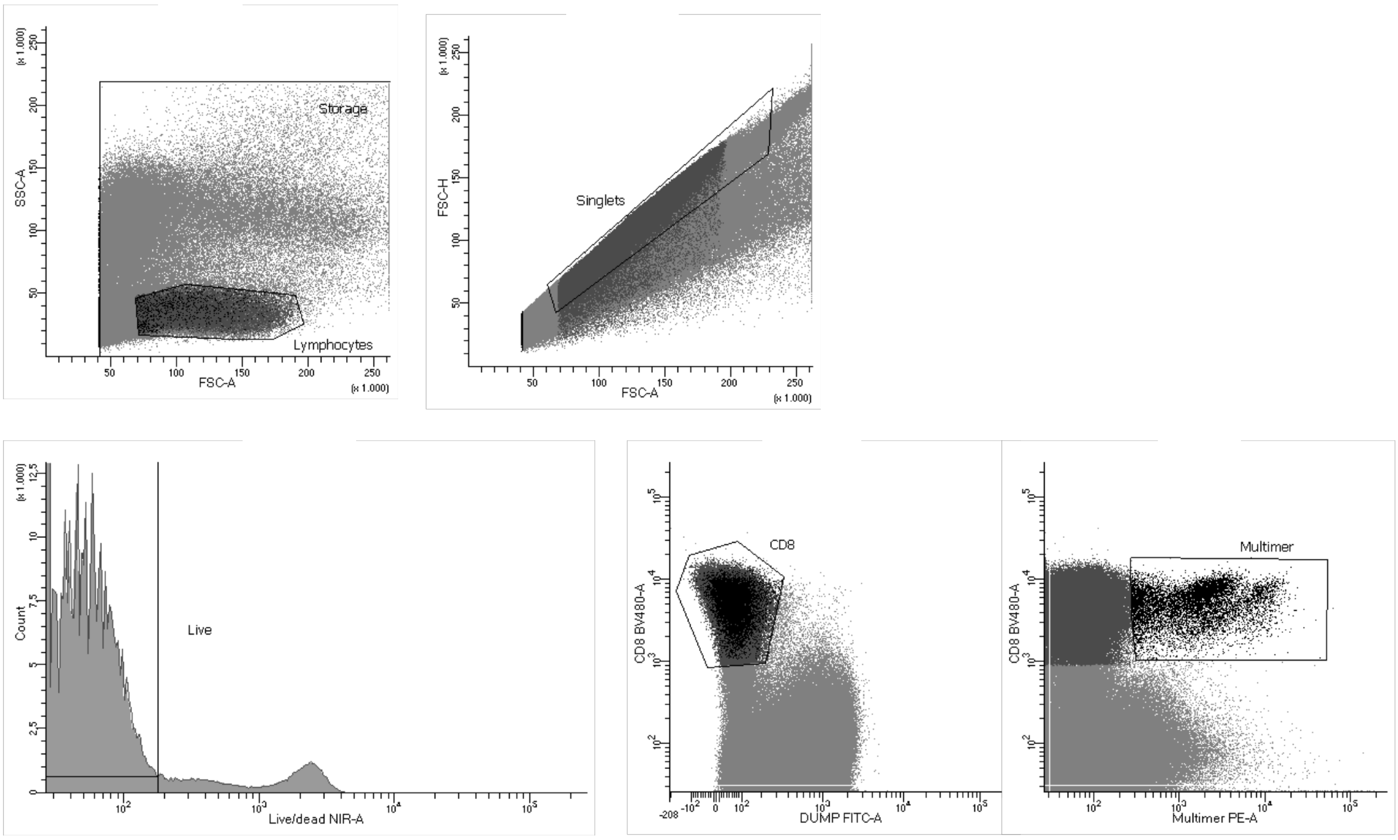

Tube: 2

Population	#Events	%Parent	%Total
All Events	2.054.539	####	100,0
Storage	2.032.190	98,9	98,9
Lymphocytes	806.906	39,7	39,3
Singlets	800.318	99,2	39,0
Live	756.090	94,5	36,8
CD8	226.527	30,0	11,0
Multimer	8.651	3,8	0,4

Supplementary Figure 1

Gating strategy. Gating strategy used to identify single, live lymphocytes and within these, CD8 and MHC multimer specific T cells. The gating strategy depicted here is applied to all experiments from which data is shown in figures 2-4.

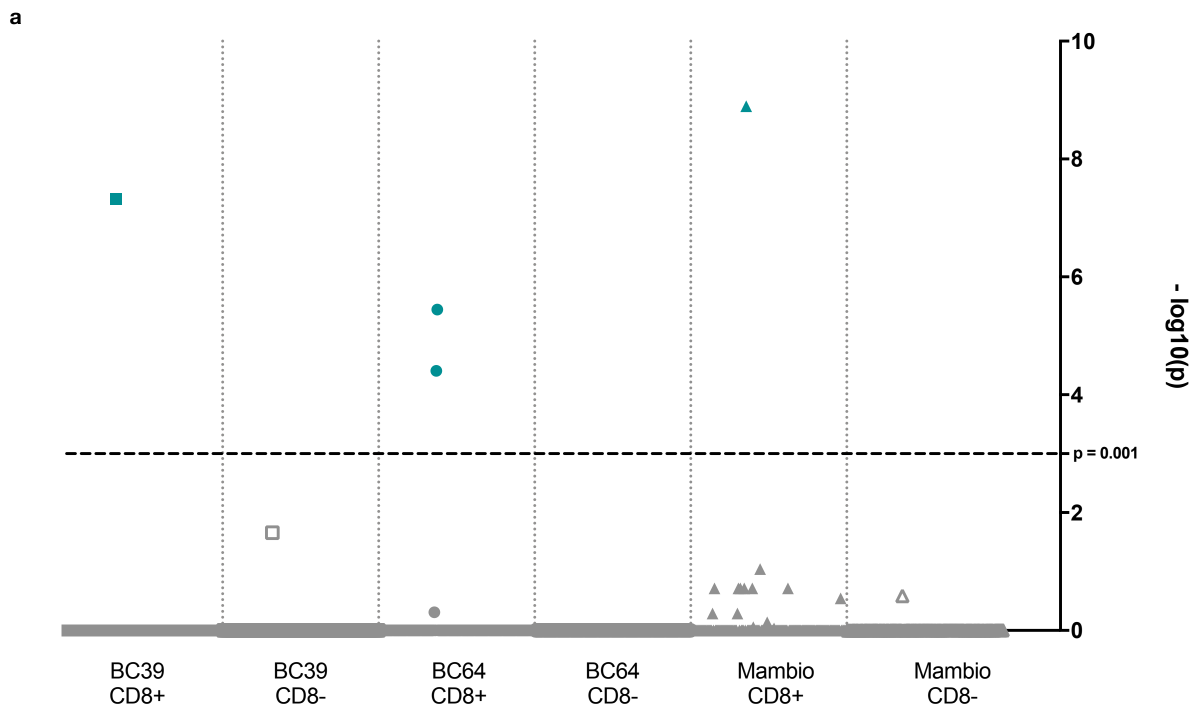

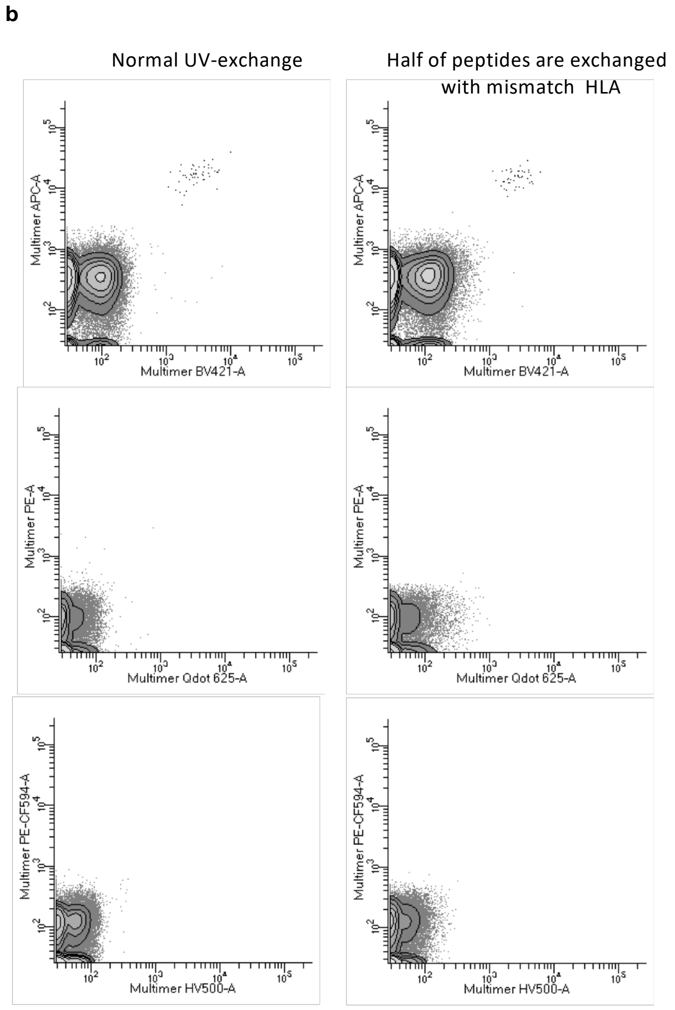

Supplementary Figure 2

Technical information related to T-cell detection using barcoded MHC multimers. **a** Virus-epitope recognition restricted to HLA-A*02:01 and HLA-B*07:02, detected in a test experiment where both CD8$^+$ multimer$^+$ cells and CD8$^-$ cells were sorted and the associated DNA barcodes were amplified and sequenced. p values from sequencing analysis are log transformed for visualization. No significant pMHC recognition is determined in the CD8 negative population. **b** Detection of virus-epitope recognition based on combinatorial fluorescence encoding using 27 different pMHC multimers, derived from UV-induced peptide exchange either using HLA-relevant virus epitopes (left panel) or in a separate panel where half of the peptides were replaced with an irrelevant HLA non-matching (non-binding) HLA-A*03:01 peptide (right panel). No significant enhancement of background was observed when half on the pMHC complexes where non-functional, unstable, pMHC combinations. BC: Healthy donor, Mambio: sample from a breast cancer patient.

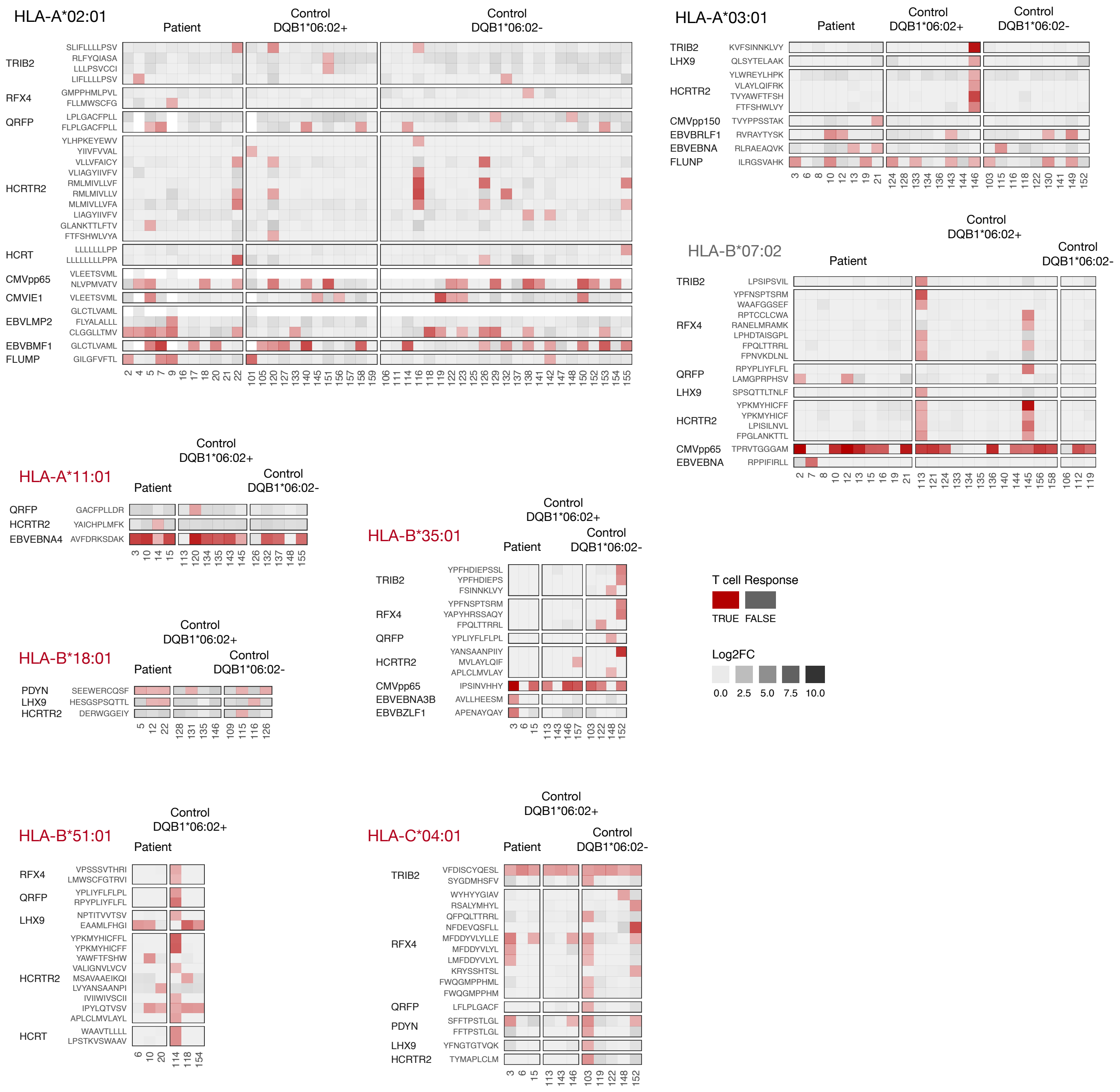

Supplementary Figure 3

Overview of CD8⁺ T cell recognition. Overview of all peptides for which CD8⁺ T cell recognition was detected and their HLA restriction. Red color indicates detected CD8⁺ T cell recognition of the given peptide within the given donor (number below the panels).

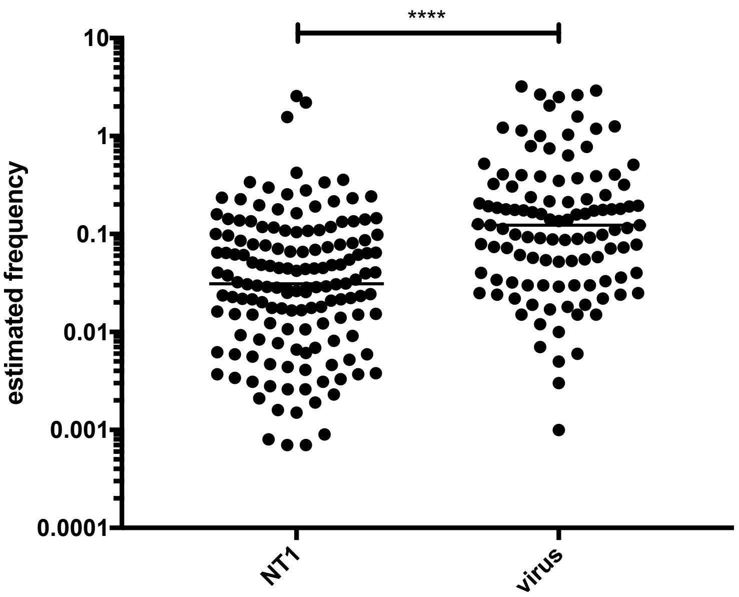

Supplementary Figure 4
NT1 and virus recognition. Estimated frequency of all CD8+ T cell populations with specificity for narcolepsy relevant peptides and virus peptides, across all donor cohorts, p<0.0001. (T test on log transformed data). Bars represent median values with 95% CI.

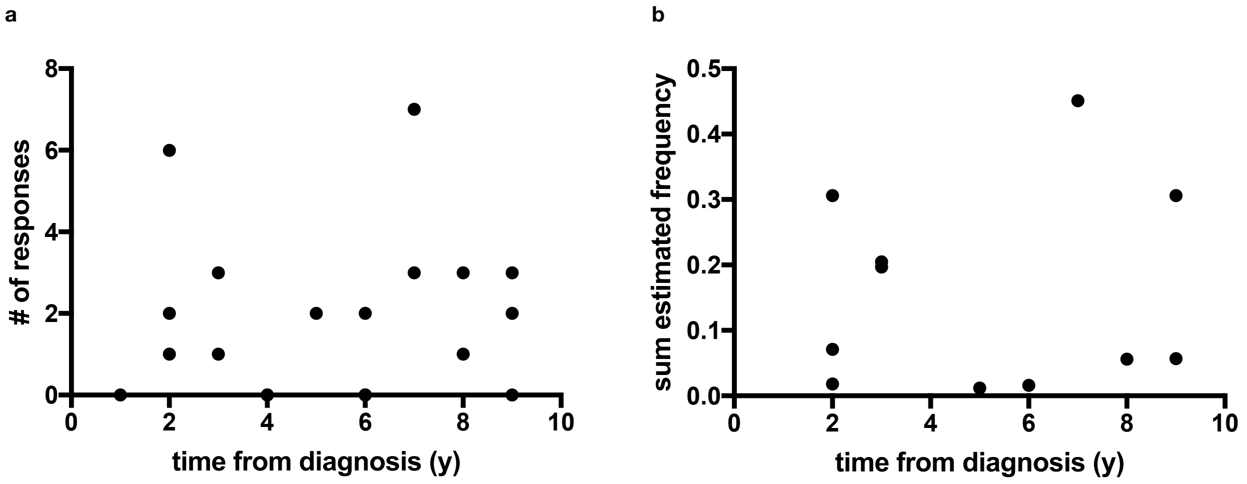

Supplementary Figure 5
Kinetics of T cell recognition. **a** The time from diagnosis of NT1 is plotted against the number of peptide-specific CD8+ T cell populations detected and b sum of estimated frequencies. Each dot represents one NT1 patient. No correlation was observed.

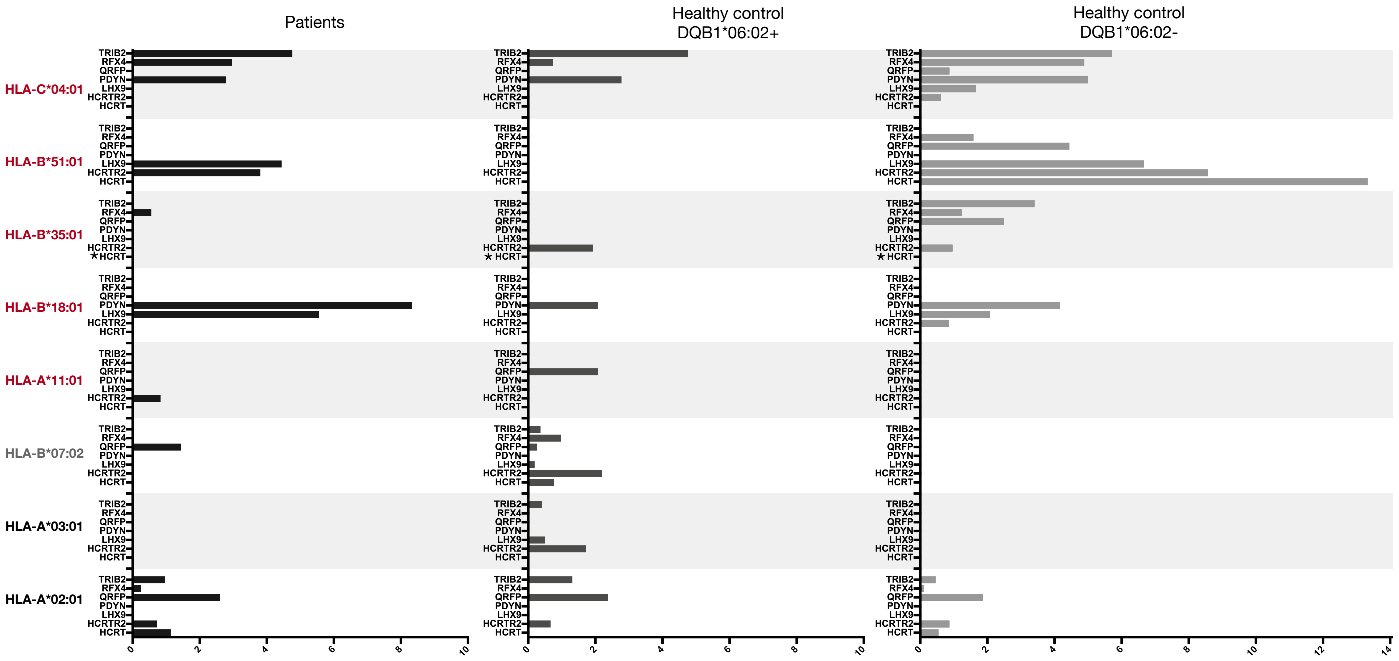

Supplementary Figure 6
The response percentage within each protein and HLA type. The number of CD8+ T cell responses within a given protein across all donors with a specific HLA type, out of the total number of possible responses (i.e. number of pMHC complexes included for that given protein and HLA combination* number of donors with the HLA type). n = the number of donors in each HLA type.

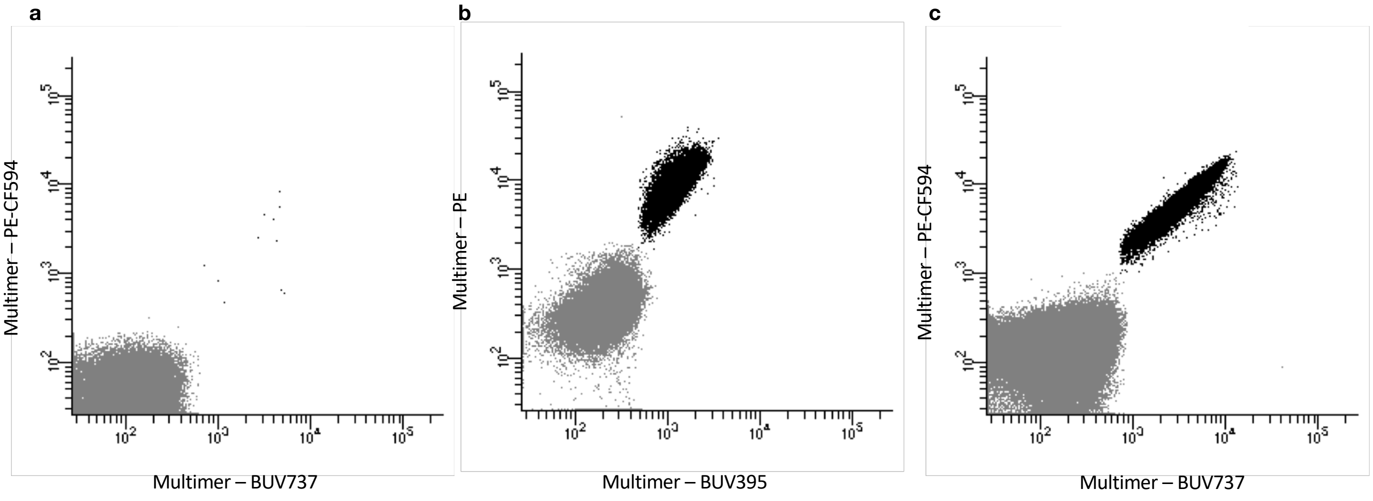

Supplementary Figure 7
Verification of T cell recognition toward an NT1-relevant peptide. **a** Dot plot showing an ex vivo MHC multimer staining for the LHX9 peptide EAAMLFHGI, binding to HLA-B*51:01 in patient 6. The EAAMLFHGI multimer was made with 2 different fluorochromes, PECF594 and BUV737 and double positive cells are pMHC specific T cells. **b** and **c** Examples of dot plots showing virus-epitope specific T cells after enrichment from a Narcolepsy patient and healthy donor respectively. PE: phycoerythrin. BUV: brilliant ultra violet.

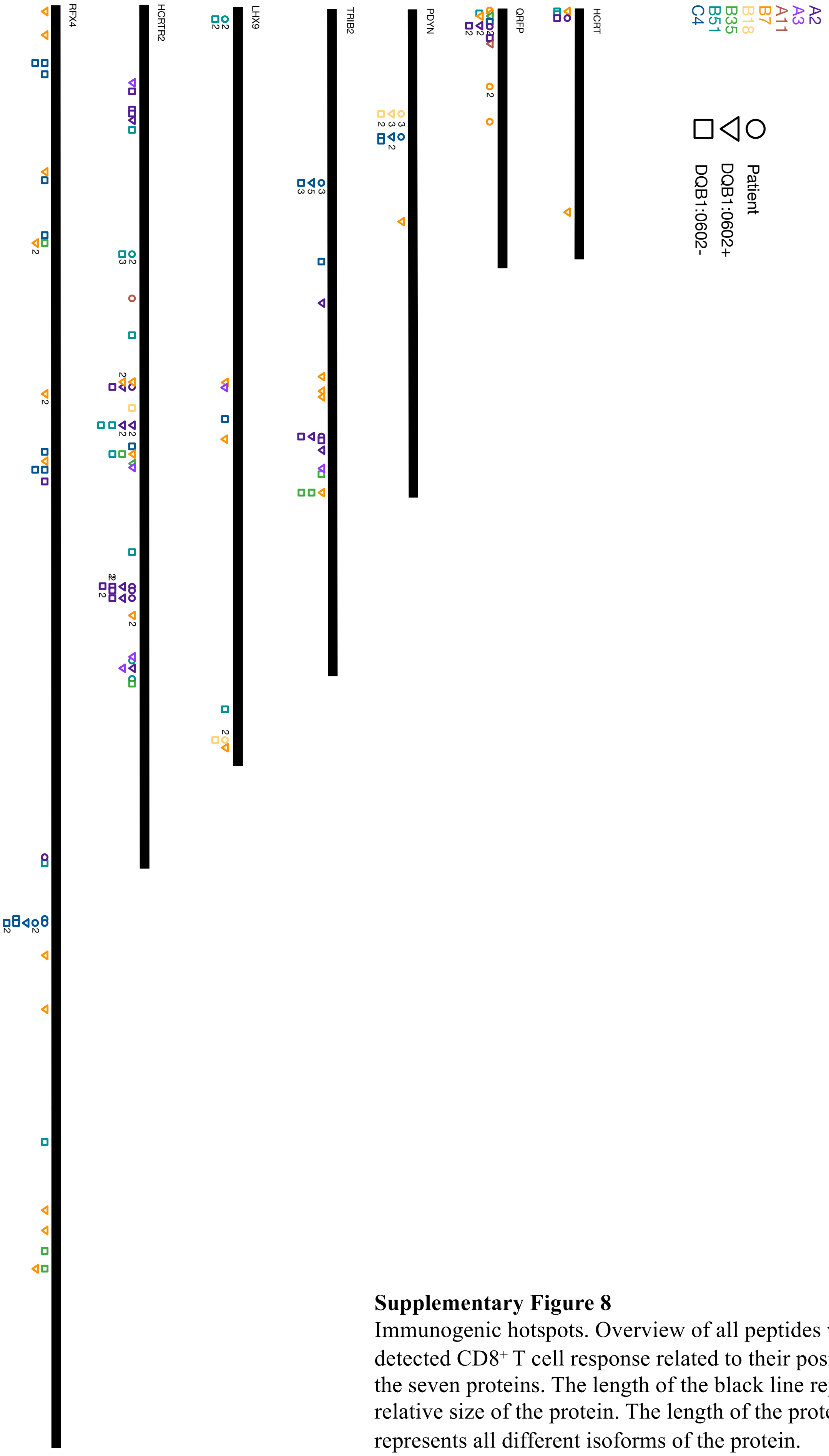

Supplementary Figure 8
Immunogenic hotspots. Overview of all peptides with a detected CD8^{+} T cell response related to their position within the seven proteins. The length of the black line represents the relative size of the protein. The length of the protein represents all different isoforms of the protein.
